# Modified Five-Flap Z-plasty for Surgical Correction of Webbed Neck Deformity in Turner Syndrome

**DOI:** 10.7759/cureus.39312

**Published:** 2023-05-21

**Authors:** Mohd Tarmizi Mohd Said, Ahmad Sukari Halim, Mohammad Ali Mat Zain, Khai Luen Koh

**Affiliations:** 1 Department of Plastic and Reconstructive Surgery, Hospital Kuala Lumpur, Kuala Lumpur, MYS; 2 Reconstructive Sciences Unit, School of Medical Sciences, Health Campus, Universiti Sains Malaysia, Kubang Kerian, MYS

**Keywords:** pterygium colli, turner syndrome, z-plasty, surgical management, webbed neck deformity

## Abstract

Webbed neck deformity is a congenital anomaly that exists in several syndromes. Various techniques for surgically correcting the webbed neck deformity have been described in the literature, each comes with its own advantages and disadvantages. The aim of surgery is to achieve normal neck contour and symmetrical hairline, avoid excessive scarring over the anterior and lateral neck, and limit recurrence. In this report, we described our experience in managing a case of Turner syndrome with bilateral webbed neck deformity using the modified five-flap Z-plasty technique.

## Introduction

Turner syndrome is a rare congenital anomaly affecting one in every 2000 live-born female infants. Affected children genotypically lack one of the X chromosomes - commonly referred to as XO - resulting in characteristic findings such as webbed neck deformity, low nuchal hairline, short stature, high arched palate, lymphedema of both hands and feet, broad chest with widely spaced nipples, scoliosis, hypothyroidism, cardiac and renal anomalies, coarctation of the aorta, ovarian dysgenesis, and cutis laxa. It was first described by Turner in 1938 based on a series of female patients presented in the classic triad of webbed neck, sexual infantilism, and cubitus valgus [1].

Webbed neck deformity, also known as ‘pterygium colli’, consists of a subcutaneous band of thickened fascia superficial to the trapezius muscle typically extends from the mastoid to the acromion process [2-4]. Apart from Turner syndrome, similar characteristics can also be seen in a number of syndromes such as Noonan syndrome, Klippel-Feil syndrome, and Escobar syndrome [2]. Although it has been recognized for over 100 years, the exact etiology is still not completely understood [2].

The main concern in dealing with the deformity is mainly aesthetic, as many children with Turner syndrome or their parents seek surgical correction of the webbed neck deformity to avoid being teased by peers especially when they reach school age, which may negatively impact their emotional and psychosocial development. The aim of surgery is to achieve normal neck contour and symmetrical hairline, avoid excessive scarring over the anterior and lateral neck, and limit recurrence. Various surgical techniques have been described in the literature to achieve the above objectives [2-6]. In this report we described our experience in managing a case of Turner syndrome with bilateral webbed neck deformity that was surgically corrected using modified five-flap Z-plasty, better known as the ‘Jumping man’ Z-plasty technique.

## Case presentation

**Figure 1:**
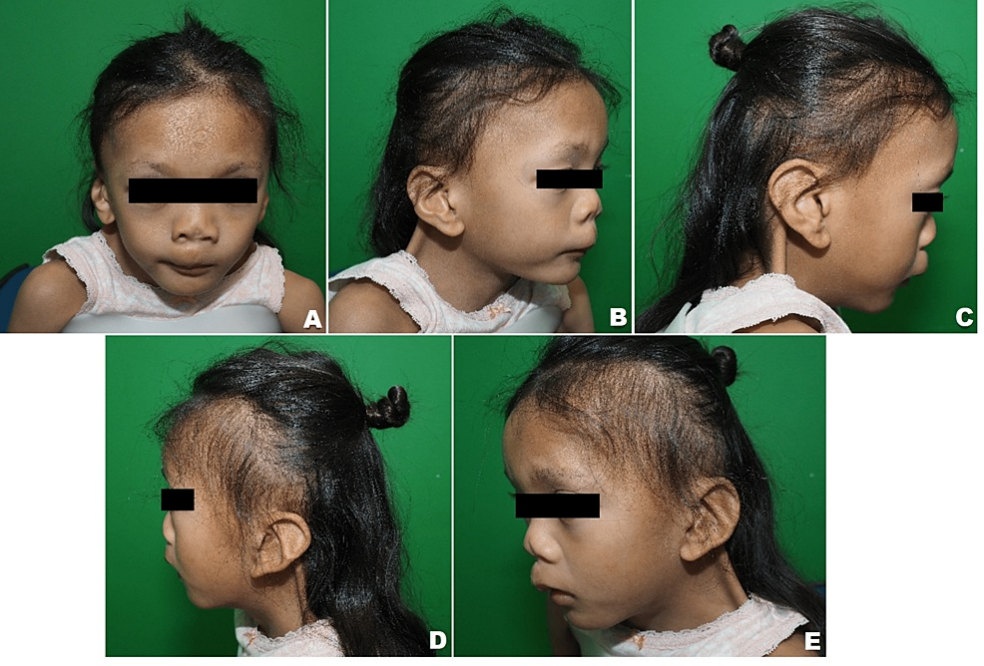
Pre-operative photo of the patient. Notice the bilateral congenital webbed neck deformity and bifid nose. (A) Anterior view (B) Right oblique view (C) Right lateral view (D) Left lateral view (E) Left oblique view

This is a five-year-old Malay girl who was referred to our department for a bilateral webbed neck and bifid nose. She was diagnosed with Turner syndrome and congenital hypothyroidism. Antenatal history was unremarkable. Her developmental milestones were generally up to her age. Clinically she has prominent webbing of the bilateral lateral neck extending from the mastoid to the acromion process (Figure 1). Apart from that she also had a bifid nose with soft tissue deficiency over the nasal tip. The main reason for referral was parental concern about the child’s appearance and keenness for surgical correction of the bilateral webbed neck as well as bifid nose prior to attending school.

**Figure 2:**
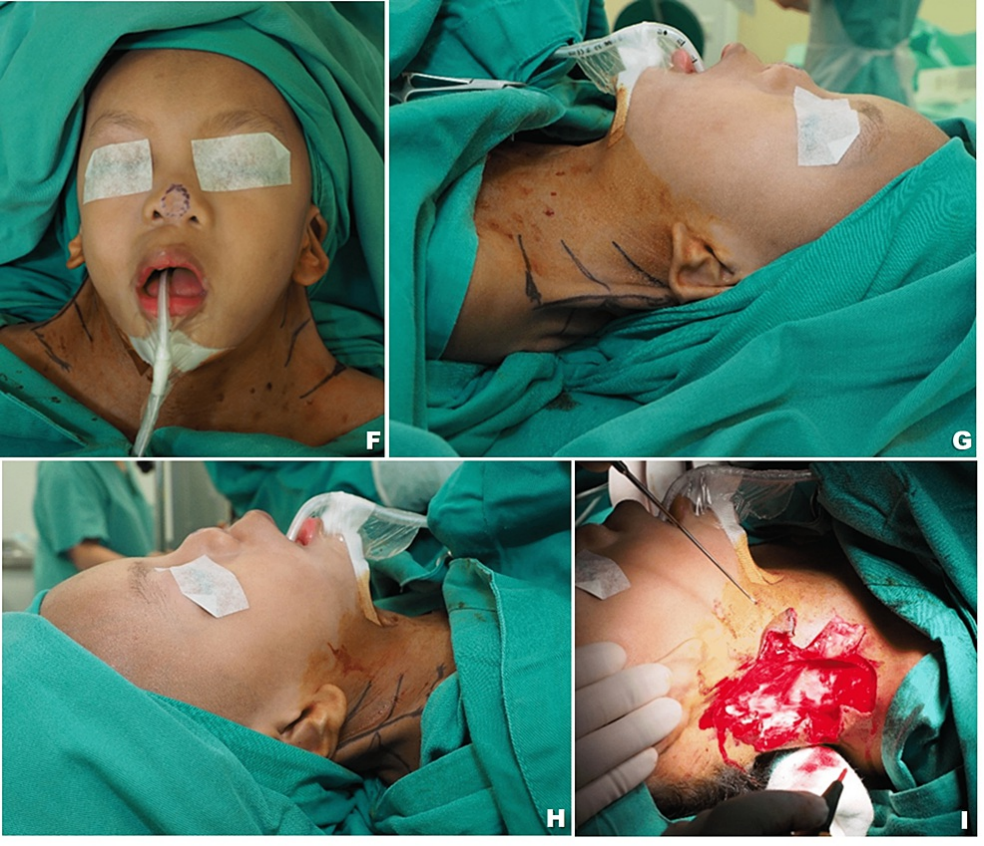
Pre-operative flap planning. Correction of bilateral webbed neck was done under the same setting. Five-flap Z-plasty was designed with two central flaps anteriorly and two lateral flaps posteriorly similar to double opposing Z-plasty. A triangular Y-V advancement flap was designed between the two opposing Z-plasties. A thickened layer of fascial band overlying the trapezius muscle can be seen following dissection. (F) Anterior view (G) Left lateral view (H) Right Lateral view (I) Intraoperative view of the right webbed neck following excision of the web

**Figure 3:**
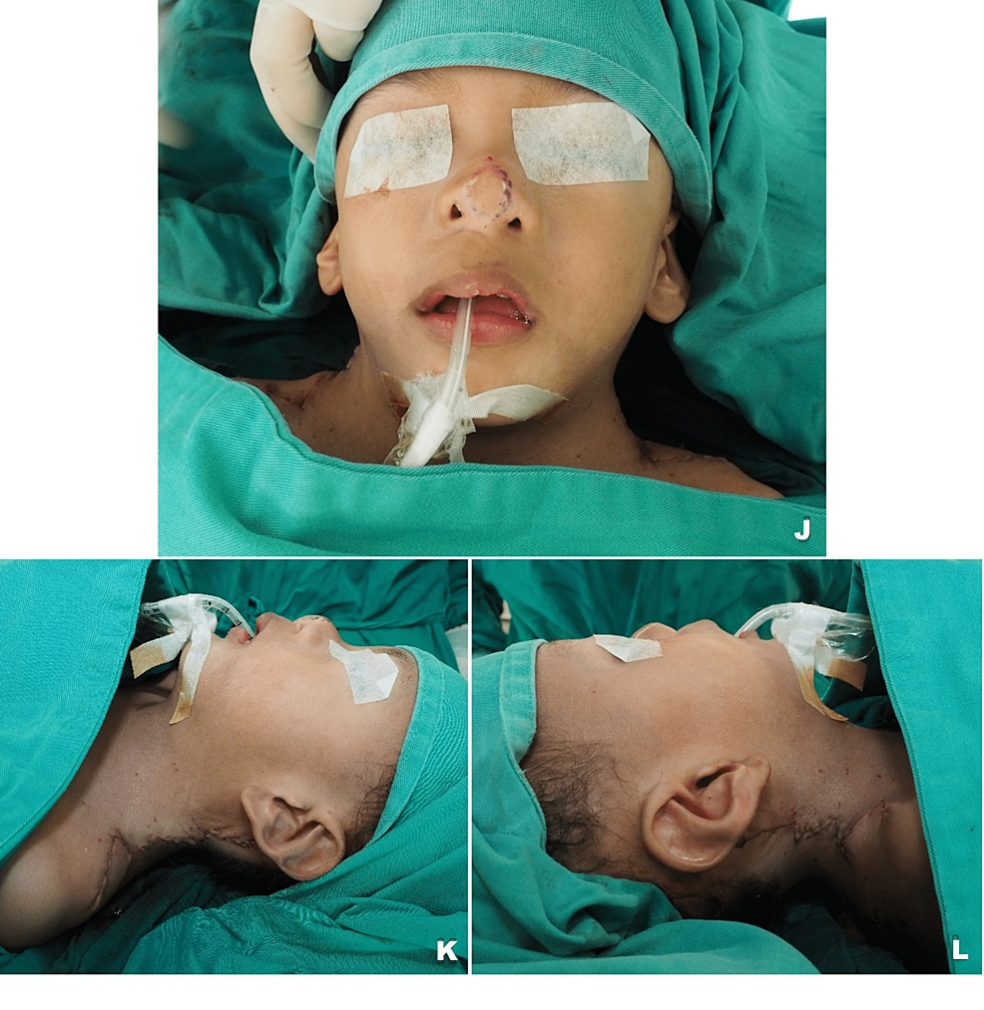
Immediate postoperative view. The anterior glabrous skin flap demonstrated laxity and was able to be transposed posteriorly, meanwhile the overlapping hair-bearing posterior skin flap was excised. The wound was closed by layers in a zig-zag manner. Notice the restoration of bilateral neck contour. (J) Anterior view (K) Left lateral view (L) Right lateral view

A formal workup and medical clearance were done before she was electively admitted for surgery. The correction of the bilateral webbed neck was done under the same setting. The patient was placed in a supine position under general anesthesia with the patient’s head slightly tilted to the contralateral side of the corrected lateral neck. The lateral approach, the five-flap Z-plasty technique was selected as the mode of surgical correction (Figure 2). The main limb was drawn along the fibrous band running from the mastoid to the acromion process. The central flap was placed anteriorly to form two anterior limbs of Z-plasty skin flaps, meanwhile the lateral flap that will form two posterior limbs of Z-plasty was placed posteriorly. In this case, an angle of approximately 60 degrees has been used for both the anterior and posterior limbs of Z-plasty. A triangular flap that was created in between the two posterior limbs was advanced anteriorly towards an incision made between the two anterior skin flaps in a Y-V fashion. This will allow a theoretically longer scar which results in the deepening of the lateral neck web. The skin flaps were raised subcutaneously, fibrous bands were separated and excised from the underlying trapezius muscle, then overlapping excess hair-bearing posterior skin flap edges were trimmed and excised as the glabrous anterior skin flaps were transposed in place posteriorly. To obliterate a dead space, the skin flaps were stitched down to the underlying investing fascia. Wound closure was done in layers in a zig-zag fashion using absorbable sutures (Figure 3).

**Figure 4:**
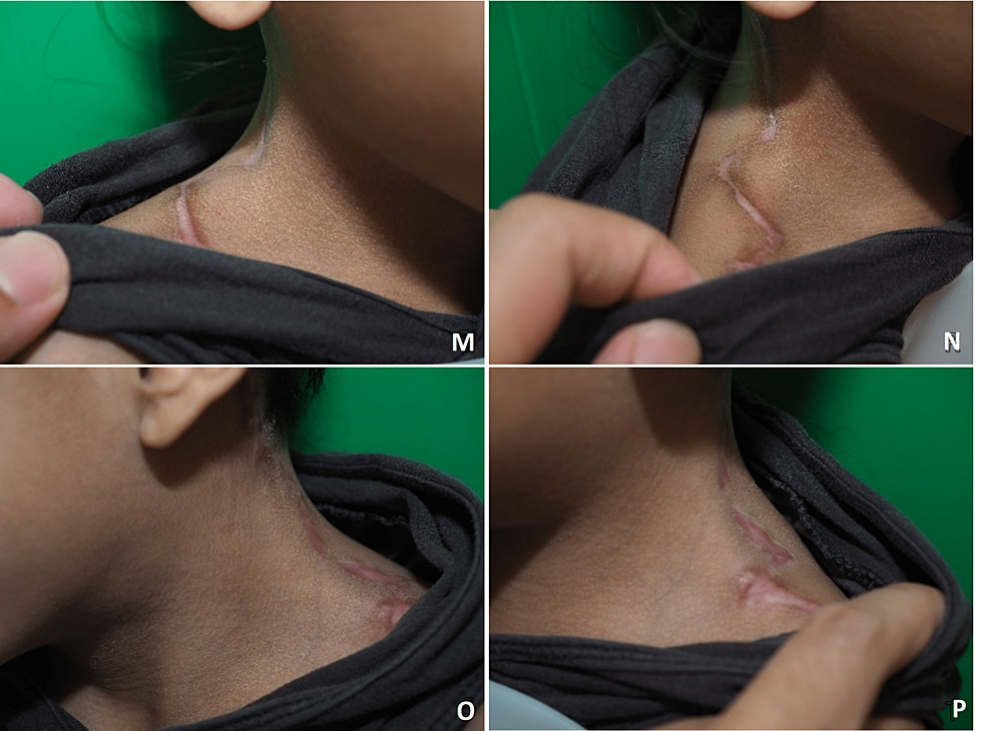
Early result three months after surgery. The contour of right side of the neck has improved with no recurrence seen when compared to the left side. The scar was slightly hypertrophic. (M, N) Scar profile of the right lateral neck (O, P) Scar profile of the left lateral neck.

The patient had an uneventful postoperative period. Upon follow-up, we noticed a mild recurrence of the left lateral neck web compared to the right side. The scar was slightly hypertrophic but managed conservatively (Figure 4). Otherwise, the parents were happy with the outcome.

## Discussion

Since its first description by Kobylanski in 1883 [7], there have been numerous techniques implemented and described in the literature to surgically correct the webbed neck deformity. The aim of surgical correction of webbed neck deformity is to correct the lateral neck contour deformity, redistribution, or removal of the horizontal excess skin, address the abnormal posterior hairline, avoid excessive scarring over the anterior and lateral neck, and limit recurrence. Surgical correction may be accomplished via lateral [2,4,5] or posterior approach [3,6].

In 1937, Chandler described the lateral approach using traditional Z-plasty to lengthen and break up the web, which satisfactorily addresses the neck contour deformity [8]. It also allows for complete visualization and excision of the fascial web. However, this technique resulted in an unnatural hairline as the unsightly hair-bearing tissue is being displaced forward, which subsequent technical modification has been proposed by Macgregor and McGregor in 1995, and Hikade et al. in 2002 to minimize the anterior transposition of the hairline. In their series, Hikade performed a lateral Z-plasty, excision of the hair-bearing flap, and addition of two Z-plasty to correct the resultant dog-ear near acromion process as the anterior Z-plasty limb being transposed towards superior and posteriorly. Visible surgical scars over the lateral aspect of the neck extending from the mastoid to the acromion process become another disadvantage of this technique, making it less favorable. With the availability of laser, scar resurfacing, and post-operative laser hair removal may be considered as a useful adjunct [3].

Menick et al. (1984) described another technique of lateral approach using a cervical advancement flap [9]. An incision made along or within the abnormal hairline from the mastoid down to the posterolateral neck. The redundant posterior hair-bearing skin is then excised. The platysma with the anterior neck skin was elevated and subsequently retracted superior and posteriorly to obliterate the fibrous web. Closure of the wound is started inferiorly which produces a dog ear on the occipital scalp, which can be corrected within the hairline. This technique is useful in elevating the low hairline and allows direct visualization of the structural anatomy of the web. In his series, no recurrences were seen at the two-year follow-up.

The posterior approach was first reported by Foucar in 1948, in which two lateral flaps were undermined through a midline incision followed by a T shape closure with a vertical limb on the posterior midline of the neck [10]. Shearin and De Franzo (1980) reported midline posterior butterfly incision similar to Foucar technique [11]. The posterior skin was stretched posteriorly in the direction of the central point until webbing is corrected. The resultant four folds of excess skin were then excised and closed in a double inverted Y. These techniques have the advantage of producing more natural hairlines with no visible anterior or lateral neck scar. However, the scar tends to be put under significant tension and spreads over time. As the fibrous band was never excised, the approach is associated with a higher recurrence rate of the web [3,4,6].

A combination of techniques has been proposed by Reichenberger et al., in which a series of 11 patients who underwent three different techniques - including Z-plasties, advancement flaps, or a combination of both - has been reviewed [2,12]. The technique was selected based on the patient’s characteristics. In patients with no abnormal hairline, Z-plasties will provide tension-free closure with good recontouring. For correction of the lateral hairline or excess skin, advancement flaps were designed with an ellipsoid excision. A combined technique practically includes ellipsoid excision, advancement flaps, and lateral Z-plasty. In their series only a patient with Z-plasty technique developed mild recurrence, meanwhile, all three patients with ellipsoid excision and advancement flaps developed mild to moderate hypertrophic scarring. They proposed that the combined technique offers the possibility to preserve the natural position of the hairline, having a lower chance for recurrence by removal of the fibrotic band through ellipsoid excision while avoiding scar contracture and functional impairment by lengthening the scar using Z-plasties.

To the author's knowledge, this is the first case encountered in our center for surgical correction of webbed neck deformity using the lateral approached five-flap Z-plasty technique. The five-flap Z-plasty is a variation of the classical Z-plasty technique that combined a double opposing Z-plasty in series with the addition of a Y-V advancement flap between the two [13]. Theoretically, it provides a total of 125% scar lengthening - 75% from double opposing Z-plasty, another 50% from Y-V advancement flap - which is useful in deepening a webspace. In our case, there was a demonstrable laxity of anterior glabrous web skin which can be advanced posteriorly, while overlapping posterior hair-bearing web skin can be discarded, allowing tension-free closure with preservation of a more natural hairline. The versatility of the technique permits the surgery to be conducted in any patient’s position, provides excellent exposure for complete excision of the thickened fascial web to prevent a recurrence, as well as allows a tension-free closure of the wound to prevent postoperative scar contracture [2,3,13,14].

## Conclusions

Various technique for surgical correction of webbed neck deformity has been described in the literature over the past century - each carries its own pro and cons. The selection of technique should be based on the patient’s characteristic, which includes the laxity of web skin and the extension of the posterior hairline. Our technique is technically easy to perform and provides reasonable advantages in improving the lateral neck contour, achieving a more natural hairline, low chance of recurrence, and providing cosmesis.
